# Collaborative intelligence for intensive care units

**DOI:** 10.1186/s13054-021-03852-7

**Published:** 2021-12-14

**Authors:** Kay Choong See

**Affiliations:** 1 Division of Respiratory and Critical Care Medicine, Department of Medicine, National University Hospital, University Medicine Cluster, National University Health System, 1E Kent Ridge Road, NUHS Tower Block Level 10, Singapore, 119228 Singapore; 2 Department of Medicine, Yong Loo Lin School of Medicine, National University of Singapore, Singapore, Singapore

## Dear Editor,

The intensive care unit (ICU) is a rich and complex data environment, well-suited for artificial intelligence (AI) and machine learning techniques. Numerous AI applications are being developed for the management of critically ill patients [1], both before and during the COVID-19 pandemic [2]. However, it remains unclear how intensive care clinicians can benefit from AI. Learning from the business literature, the concept of *collaborative intelligence* can help to clarify how humans can synergize with AI [3]. For the ICU, three domains can be identified where AI can augment the human clinician, and vice versa (Table 1).

**Table 1:** Collaborative intelligence for intensive care units

Domain	How AI can augment human clinicians	How human clinicians can augment AI
Accountability and risk mitigation	Decrease risk of human fatigue by assisting with continuous and rapid multi-channel monitoring, data harvesting, organization and analysis Provide support for human decisions	Provide stress testing and simulated adversarial attacks on AI systems Provide safety netting for potentially biased algorithms Provide medicolegal support for AI tools
Sense-making	Demonstrate clinical, laboratory and radiological features contributing to an AI-driven result Provide support for human decisions by computing probabilities for the range of possible diagnoses, outcomes and actions	Provide explanations for AI-driven results to colleagues, patients and family members
Performance augmentation	Provide clinical tools for diagnosis, risk prediction, triage, treatment and other forms of decision-making Compress data into interpretable summaries Demonstrate data relationships with visualization Provide automated evidence-based medicine search and summaries Provide automated guidance during imaging procedures	Feed AI with multisource data Feed AI with relational information Provide examples of human decision-making for training of AI models Provide support for the spread of digital resources—including education of healthcare workers in digital literacy—for AI implementation and scalability

AI, Artificial intelligence

The first domain is related to *accountability and risk mitigation*, where AI amplifies human cognition while humans sustain AI [3]. The speed and consistency of digital systems allow AI to help humans perform continuous and rapid multi-channel monitoring, data harvesting, organization and analysis. Such abilities are particularly useful in the ICU, where critically ill patients quickly amass large quantities of clinical data, and clinicians are at risk of monitoring fatigue. Furthermore, AI-driven decisions can help corroborate human decisions. In return, humans can provide real-world stress testing of AI systems, including simulated adversarial attacks, which are intentional contamination of data aimed at causing AI malfunction. Additionally, humans can audit AI algorithms for accuracy and bias, enhancing confidence and trust in AI.

The second domain is related to *sense-making*, where AI interacts with humans in intelligible ways (i.e., explainable AI [4]) while humans help explain AI [3]. Rather than merely providing an output that substantiates human answers, AI can produce lists of salient features and probabilities for various diagnoses, predictions and actions, helping humans prioritize and justify decisions. To avoid the “black-box” effect, human clinicians can augment AI outputs by helping interpret these to lay-persons.

The third and final domain is *performance augmentation*, where AI embodies human skills while humans train AI [3]. Real-time AI-powered ultrasound systems to guide novices in image acquisition and interpretation are commercially available, e.g., Caption AI (Caption Health, Brisbane, CA). At the cognitive level, just like how human players train using computer chess engines, human clinicians can learn from AI-generated decisions and data summaries. Graph data science methods using multivariate time series can reveal novel visual relationships among patient characteristics, treatments and clinical evolution [5]. In turn, AI methods like reinforcement learning depend on real-life data and decision-making. Ultimately, implementation and scaling of AI solutions require human support for digital resources.

## Data Availability

Not applicable.

## Abbreviations

AI: Artificial intelligence
ICU: Intensive care unit
